# Maternal and Neonatal Prognostic Factors for Cardiorespiratory Events in Healthy Term Neonates During Early Skin-to-Skin Contact

**DOI:** 10.3389/fped.2022.907570

**Published:** 2022-05-31

**Authors:** Jesús Rodríguez-López, Javier De la Cruz Bértolo, Nadia Raquela García-Lara, Izaskun Asla Elorriaga, Lourdes Román Echevarría, Máximo Vento, Anna Parra-Llorca, Fernando Cabañas, Pedro Lozano, Óscar García-Algar, Ana Martín-Ancel, Cristina Copons Fernández, Ersilia González Carrasco, Iciar Olabarrieta Arnal, Adelina Pellicer, Natalia Marín Huarte, Carmen Rosa Pallás-Alonso

**Affiliations:** ^1^Department of Neonatology, Health Research Institute Imas12, 12 de Octubre University Hospital, Complutense University, Madrid, Spain; ^2^Health Research Institute Imas12, 12 de Octubre University Hospital, Madrid, Spain; ^3^Neonatology Unit, Cruces University Hospital, Baracaldo, Spain; ^4^Division of Neonatology, Neonatal Research Group, Health Research Institute La Fe, University and Polytechnic Hospital La Fe, Valencia, Spain; ^5^Department of Pediatrics and Neonatology, Quirónsalud Madrid University Hospital, Madrid, Spain; ^6^Neonatology Unit, Institut Clínic de Ginecologia, Obstetrícia i Nonatologia (ICGON), Institut d’Investigacions Biomèdiques August Pi i Sunyer (IDIBAPS), Hospital Clinic-Maternitat, Barcelona, Spain; ^7^Neonatology Unit, Sant Joan de Déu University Hospital and Clínic University Hospital, BCNatal, Barcelona, Spain; ^8^Department of Neonatology, Vall d’Hebron University Hospital, Barcelona, Spain; ^9^Neonatology Unit, Severo Ochoa University Hospital, Madrid, Spain; ^10^Department of Neonatology, La Paz University Hospital, Madrid, Spain

**Keywords:** early skin-to-skin contact, oxygen desaturation, healthy term newborns, sudden unexpected postnatal collapse (SUPC), oxygen saturation monitoring, prognostic factor, cardiorespiratory events, pulse oximetry

## Abstract

**Background:**

During early skin-to-skin contact (ESSC), alterations in peripheral oxygen saturation (SpO_2_) and heart rate (HR) have been frequently observed.

**Objectives:**

This study aimed to determine the incidence of cardiorespiratory events (CREs) during ESSC in healthy term newborns (HTNs) and estimate the association of maternal and neonatal prognostic factors with the risk of CREs.

**Methods:**

A pooled analysis of the cohort from a clinical trial involving healthy mother–child dyads during ESSC was performed. Pulse oximetry was employed to continuously monitor SpO_2_ and HR within 2 h after birth. The individual and combined prognostic relevance of the demographic and clinical characteristics of dyads for the occurrence of a CRE (SpO_2_ <91% or HR <111 or >180 bpm) was analyzed through logistic regression models.

**Results:**

Of the 254 children assessed, 169 [66.5%; 95% confidence interval (95% CI), 60.5–72.5%] had at least one CRE. The characteristics that increased the risk of CRE were maternal age ≥35 years (odds ratio, 2.21; 95% CI, 1.19–4.09), primiparity (1.96; 1.03–3.72), gestational body mass index (BMI) >25 kg/m^2^ (1.92; 1.05–3.53), and birth time between 09:00 p.m. and 08:59 a.m. (2.47; 1.02–5.97).

**Conclusion:**

CREs were more frequent in HTNs born during nighttime and in HTNs born to first-time mothers, mothers ≥35 years, and mothers with a gestational BMI >25 kg/m^2^. These predictor variables can be determined during childbirth. Identification of neonates at higher risk of developing CREs would allow for closer surveillance during ESSC.

## Introduction

Early skin-to-skin contact (ESSC) in the first hours postpartum provides clear benefits for both the mother and newborn (1–4) and it is now a generalized practice. An increase in the reporting of sudden unexpected postnatal collapse (SUPC) cases during ESSC has been observed (5–8). In 2017, the World Health Organization estimated an incidence between 1.6 and 5 cases per 100,000 live births during the first 2 h after delivery. Moreover, 50% of infants with a SUPC during ESSC died, and up to 50% of survivors had neurological sequelae (6, 9–11).

The etiology of SUPC is unknown (12, 13). To date, studies on SUPC are limited to case series. Although the risks of SUPC have not been estimated, the following related factors have been identified: primiparous mothers; mothers with a body mass index (BMI) >25 kg/m^2^; mothers who have used medications that cause drowsiness or sedation, tiredness, and sleepiness after childbirth; newborn placed in a prone position over the mother’s body; breastfeeding; absence of surveillance by a companion or health personnel; and the mother being distracted by electronic devices (mobile phone, etc.) particularly during ESSC (4–6, 12–17). Similarly to the sudden infant death syndrome, studies on the causes of SUPC are difficult to conduct. However, oxygen desaturation episodes during ESSC within the first 2 h after delivery are extremely common in healthy term newborns (HTNs) (18). By identifying the risk factors of cardiorespiratory events (CREs), it may also be possible to identify the HTNs with the highest risk of SUPC and enhance surveillance measures for them.

This study aimed to determine the incidence of CREs during ESSC in HTNs within 2 h after delivery and estimate the association of maternal and neonatal prognostic factors with the risk of CREs. The definition of factors that increase the risk of CREs would allow to identify mother–child dyads that may require more rigorous clinical control and monitoring using a pulse oximeter.

## Materials and Methods

### Data Source

In this study, we analyzed the data collected from the participants of the two arms of a multicenter, randomized, and controlled clinical trial pooled in a single cohort. The trial was designed to estimate the effect of two different positions of the mother’s bed, at 45° with respect to the horizontal axis versus 15°, on the occurrence of oxygen desaturation (SpO_2_ <91%) in HTNs during ESSC within 2 h of birth (18). The study was part of the work program of the Spanish Network for Collaborative Research in Maternal and Child Health (SAMID Network). Ten Spanish tertiary-level hospitals were selected, and the coordinating center was the Madrid University Hospital 12 de Octubre. The trial was approved by the Clinical Research Ethics Committee of all participating centers. The dyads were recruited between November 2015 and June 2018. The study was registered in ClinicalTrials.gov under number NCT025854929.

### Participants

**FIGURE 1:**
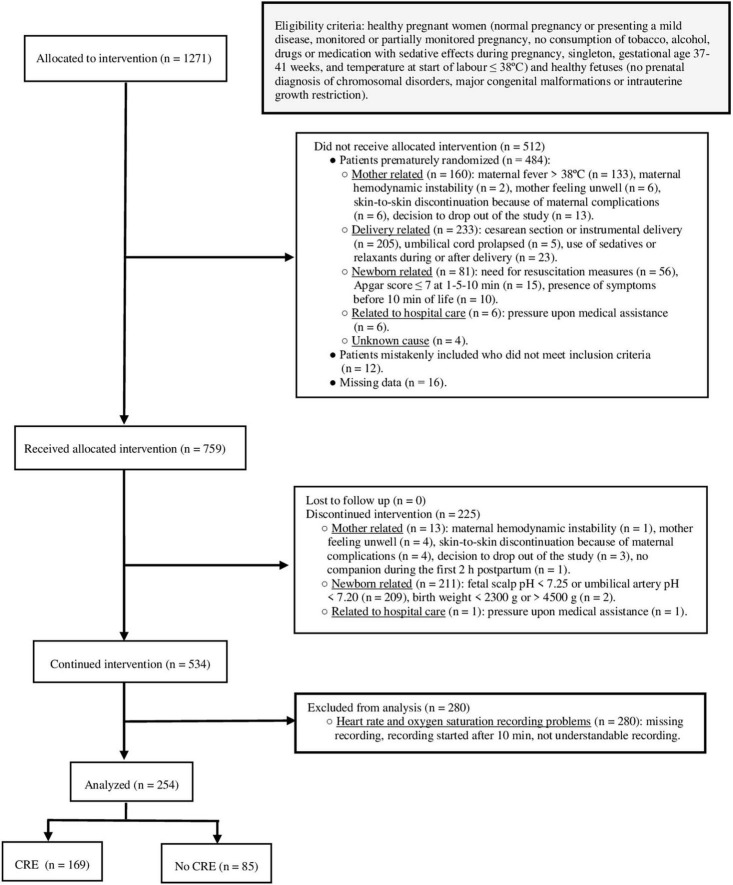
Study flow diagram. Eligibility and post-randomization exclusion criteria. Participants with and without outcome. CRE, cardiorespiratory event.

We aimed to enroll potentially eligible mothers admitted to 10 Spanish obstetric units and include mother–child dyads of HTNs and mothers without significant medical history. The eligibility criteria were checked at the start of labor. The pregnant women had normal pregnancy or mild disease [gestational diabetes treated exclusively with diet or arterial hypertension (with no diagnosis of preeclampsia) controlled with a single drug], monitored or partially monitored pregnancy, singleton, full-term pregnancy, temperature ≤38°C, presence of a companion during the first 2 h after delivery, and mother’s wish for ESSC. The pre-randomization exclusion criteria were as follows: consumption of tobacco, alcohol, drugs, or medication with sedative agents during pregnancy; moderate/severe disease during pregnancy; prenatal diagnosis of chromosomal disorders; major congenital malformations; and intrauterine growth restriction. Likewise, clinical situations involving the mother or newborn during the birth process that lead to instability and limit starting ESSC, impossibility of initiating monitoring due to excessive researcher workload, and the presence of problems with SpO_2_ and heart rate (HR) data registration were reasons for post-randomization exclusion (Figure 1).

### Outcome

The primary endpoint of this study was the occurrence of a first CRE, defined as an episode of SpO_2_ <91% or HR <111 or >180 beats per minute (bpm) in an asymptomatic HTN, unexplained by a technical reason (poor signal) or the newborn’s condition (crying, newborn handled by the mother, and movement artifacts).

The procedure of the initial trial involved continuous monitoring of preductal SpO_2_ and HR in the newborns by pulse oximetry, from 10 min to 2 h after delivery, while they underwent ESSC with their mother.

### Data Collection and Processing

The initial trial involved investigators’ training sessions by the coordinating center in all participating centers (19). The inclusion of each participant was confirmed by the coordinating center. Maternal, gestational, childbirth, and neonatal demographic data were prospectively and locally collected in hospitals and stored in the central marsupiNote database.^1^ CRE data were extracted from a pseudonymized Excel file containing the pulse oximetry records. The study include both a local and central reading of the SpO_2_ and HR records. Central reading was independent of maternal and neonatal characteristics. In case of discrepancy, incorrect or absent data, the coordinating center contacted and discussed each case with the study centers and established the final assessment.

### Predictors

The following maternal and neonatal characteristics were considered: maternal age, maternal country of birth, relevant medical history, parity, previous child who died within the first year of life, intention to breastfeed, gestational BMI, medication consumption within 72 h before delivery, delivery onset, use of epidural analgesia, time of membrane rupture, delivery duration, maternal medication during labor, gestational age, birth time, ≥1 nuchal cord, infant sex, 1-min Apgar score, 5-min Apgar score, birth weight, and maternal medication within 2 h after delivery.

### Sample Size

The minimum sample size calculated to estimate a one-third reduction between study arms in the number of HTNs with at least one occurrence of SpO_2_ <91% was 222 mother–infant dyads (18). The effective sample size for this study was 254 with 169 CREs. Considering a target “events per variable ratio” of 10, the recommended maximum number of candidate predictors to be assessed in the multivariable models was 9.

### Statistical Analysis

To describe the maternal and neonatal characteristics, means and standard deviations (SDs) were used for continuous variables and absolute and relative frequencies for categorical variables. Unadjusted and adjusted odds ratio (OR) and 95% confidence intervals (95% CIs) were estimated for the association between dyad characteristics and CREs through logistic regression models. Variable selection for the multivariable model was based on SUPC risk factors referenced in the literature, in addition to maternal age.

Statistical analysis was conducted using SAS/STAT software version 9.4 (SAS Institute Inc., Cary, NC, United States).

## Results

During the study period, 1271 mother–child dyads were recruited. A total of 254 dyads (20%) were included in the analysis. Figure 1 presents the participant flowchart for the study.

Of the women, 46% (116/254) were aged ≥35 years. Two-thirds of the mothers were born in Spain (164/253, 65%). Moreover, 36% (91/252) were primiparous. Gestational BMI was >25 kg/m^2^ in 66% (142/214) of the mothers. Most women (237/253, 94%) intended to breastfeed. Twenty percent (49/250) of the mothers reported having consumed medication 72 h before delivery. Labor was induced in one-fifth of the women (54/253, 21%). Furthermore, 82% (209/254) of the mothers received epidural analgesia. Delivery duration was <16 h in 97% (236/243) of the cases. One-fifth of the women were administered drugs during or within 2 h of delivery (47/246, 19%). The mean gestational age at the time of delivery was 39.2 (SD, 1.2) weeks, and the mean newborn weight was 3.314 (SD, 400) g. There were 125 (49%) male newborns. Eighteen percent (45/254) of births occurred between 09:00 p.m. and 08:59 a.m.

Two-thirds of HTNs had at least one CRE (169/254; 66.5%; 95% CI, 60.5–72.5%) within 2 h after delivery during ESSC. The median time from birth up to the first event was 30 min (IQR, 14–51). Moreover, 82% of CREs occurred within the first hour after birth. None of the CREs were accompanied by clinical symptoms or signs.

Tables 1, 2 show the result of the univariate analysis of maternal and neonatal characteristics for CREs.

**TABLE 1:** Maternal characteristics: association with cardiorespiratory event (univariable analysis).

Variables	CRE *n* = 169 (%)	No CRE *n* = 85 (%)	Odds ratio (95% CI)	*p*-Value
Age ≥35 years	83/169 (49)	33/85 (39)	1.52 (0.89–2.58)	0.121
Country of birth, Spain	109/168 (65)	55/85 (65)	1.00 (0.58–1.74)	0.978
Relevant medical history	49/163 (30)	31/83 (37)	0.72 (0.41–1.26)	0.249
Primiparous	67/168 (40)	24/84 (29)	0.60 (0.34–1.06)	0.078
Previous child who died in the first year of life	1/168 (0.6)	1/84 (1)	0.49 (0.03–8.05)	0.616
Intention to breastfeed	162/169 (96)	75/84 (89)	2.78 (0.99–7.74)	0.043
Gestational BMI >25 Kg/m^2^	95/133 (71)	47/81 (58)	1.81 (1.01–3.23)	0.045
Medication consumption during 72 h prior to delivery	22/166 (13)	27/84 (32)	0.32 (0.17–0.61)	<0.001
Non-opioid analgesics/anti-inflammatory drugs	6/166 (4)	9/84 (11)		
Thyroid hormone	4/166 (2)	10/84 (12)		
Ranitidine/almagate	3/166 (2)	7/84 (8)		
Other medications	10/166 (6)	4/84 (5)		
Induction of labor	37/168 (22)	17/85 (20)	0.88 (0.46–1.69)	0.711
Epidural analgesia	134/169 (79)	75/85 (88)	0.51 (0.24–1.09)	0.078
Rupture of membranes (<18 h)	151/169 (89)	77/84 (92)	1.31 (0.53–3.27)	0.561
Duration of delivery (<16 h)	160/165 (97)	76/78 (97)	1.19 (0.23–6.26)	0.839
Maternal medication during labor and 2 h afterward	30/165 (18)	17/81 (21)	0.86 (0.45–1.67)	0.663

*CRE, cardiorespiratory event; CI, confidence interval; BMI, body mass index.*

**TABLE 2:** Neonatal characteristics: association with cardiorespiratory event (univariable analysis).

Variables	CRE*n* = 169 (%)	No CRE *n* = 85 (%)	Odds ratio (95% CI)	*p*-Value
Gestational age 37–38 versus 39–41 weeks	46/169 (27)	30/85 (35)	0.69 (0.39–1.19)	0.185
Birth time between 09:00 p.m. and 08:59 a.m.	36/169 (21)	9/85 (11)	0.44 (0.20–0.96)	0.035
≥1 nuchal cord	41/168 (24)	21/83 (25)	0.95 (0.52–1.75)	0.877
Male	84/169 (50)	41/84 (49)	1.04 (0.61–1.75)	0.893
1-min Apgar score 8 versus 9–10	34/169 (20)	19/85 (22)	0.87 (0.46–1.65)	0.679
5-min Apgar score 9 versus 10	26/169 (15)	8/85 (9)	0.57 (0.25–1.32)	0.188
Birth weight <3,000 versus ≥3,000 g	121/169 (72)	60/84 (71)	0.99 (0.56–1.77)	0.977

*CRE, cardiorespiratory event; CI, confidence interval.*

**FIGURE 2:**
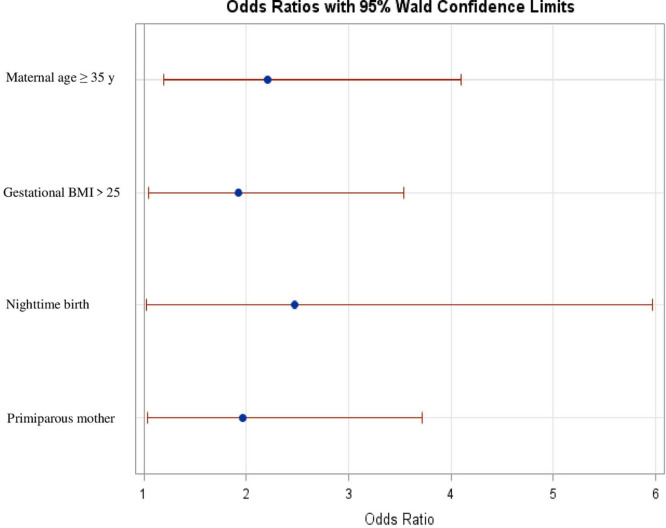
Factors associated with cardiorespiratory events (multivariate logistic regression analysis). Gestational BMI >25, gestational body mass index >25 Kg/m^2^.

On multivariable, the risk of CRE increased in mothers aged ≥35 years (OR, 2.21; 95% CI, 1.19–4.09), first-time mothers (OR, 1.96; 95% CI, 1.03–3.72), mothers with gestational BMI >25 kg/m^2^ (OR, 1.92; 95% CI, 1.05–3.53) and those whose delivery occurred between 09:00 p.m. and 08:59 a.m. (OR, 2.47; 95% CI, 1.02–5.97) (Figure 2). The variable maternal intention to breastfeed, when included in the multivariable model, was not associated with an increased risk of CRE (OR, 2.50; 95% CI, 0.74–8.43).

## Discussion

In this study, we observed that CREs more frequently occur in HTNs born during nighttime and to first-time mothers, mothers ≥35 years, and mothers with a gestational BMI >25 kg/m^2^. These characteristics are easy to recognize during the birth process and can therefore be helpful in identifying early HTNs that may have an increased risk of CREs. It should be noted that risk factors identified for CREs, except maternal age, have also been associated with SUPC. This is the first study to identify factors associated with CREs during ESSC. By identifying newborns at higher risk of developing CREs, some types of surveillance strategy could be implemented to detect possible early SUPC.

In this study we were able to assess several factors that have been related with SUPC in the literature (4–6, 12–16). We observed that all of them were associated with CREs in our study. For example, maternal primiparity was associated with SUPC and was also a predictor for CRE based on our analysis (4, 6, 12–16, 20–28). It is believed that first-time mothers are more inexperienced in proper positioning, observing, and recognizing warning signs in their child during ESSC, and breastfeeding. Some authors (20, 24) have pointed out that SUPC is more common when birth occurs during nighttime. It is possible that surveillance of the dyad is reduced during nighttime. In our study, birth between 09:00 p.m. and 08:59 a.m. was associated with an increased risk of CRE occurrence. In terms of maternal age, the proportion of children who had a CRE was higher among mothers aged ≥35 years. In the reviewed medical literature, most SUPC cases did not document the mother’s age (20–24, 28). In two studies (25, 26) that did include maternal age, of nine SUPC cases, only one mother was aged ≥35 years. The reason for the higher incidence of CREs in children of older mothers is unknown. Maternal BMI >25 kg/m^2^ has been considered by some researchers (12–15, 22, 25) as a factor favoring airway obstruction and, therefore, SUPC. In our study, a maternal BMI >25 kg/m^2^ was also associated with an increased risk of developing a CRE. Unfortunately, it was not possible to analyze some factors that have been identified as risk factors for SUPC in this study. The first of these is ESSC, which has been widely associated with SUPC. In our study, ESSC was a mandatory inclusion criterion for participation. In contrast, the absence of a companion and administration of sedative medication to the mother have also been acknowledged as risk factors for SUPC. However, both were exclusion factors for this study since we aimed to evaluate newborns in ideal conditions. Breastfeeding timing has also been linked to SUPC. However, our study did not document breastfeeding in newborns who did not have CRE; therefore, we were unable to estimate the risk associated with breastfeeding. We did analyze the intention to breastfeed communicated at the start of labor. In the univariate analysis, we identified an increased risk of CRE in women with intention of breastfeeding, although it was not confirmed when included in the multivariable model.

The finding that up to two-thirds of HTNs during ESSC had a CRE suggests that these may simply be physiological events occurring during adaptation to extrauterine life. However, some of these CREs may precede SUPC. It is also interesting to note that none of the CREs observed in our study was accompanied by clinical symptoms or signs. Conversely, in an attempt to make ESSC safer and reduce the risk of SUPC, along with close clinical surveillance, maternity wards have implemented different measures (4, 9, 10, 12–14, 16, 29–38). One of these is continuous monitoring of the newborn by pulse oximetry. Monitoring changes in SpO_2_ or HR through pulse oximetry would allow early detection of changes in respiratory and circulatory dynamics before SUPC occurs (11). In this regard, it should be noted that there is no consensus on the use of pulse oximetry during ESSC (4, 11, 12, 16, 35–37, 39). Most researchers believe that it could interfere with the development of the mother–infant bond (10, 16, 34, 37, 40). A minority (7, 11, 16, 29, 41) considers it suitable only in circumstances, such as absence of a companion and work overload at the maternity ward. Very few (7, 39, 42) recommend it as a monitoring technique during ESSC within 2 h postpartum, which is a period of greater risk of SUPC. According to the latter authors, pulse oximetry increases safety and is well valued by mothers and health personnel, and mothers do not consider it as an interference with the bonding process.

Given the controversy regarding the use of pulse oximetry, the observed association between maternal and neonatal factors easily assessed before or during the birth process and the risk of CRE allows us to argue that there is a population of HTNs that, during ESSC, could have a higher risk of such events. Perhaps, it would be advisable for this group of HTNs to be monitored more closely during ESSC. This surveillance could be clinical, by the health personnel and the mother’s companion, and through a continuous monitoring system, such as pulse oximetry.

Previous studies relate changes in SpO_2_ and HR within the first minutes of life with the type of delivery, gestational age at birth, time of cord clamping, immediate placement or non-placement of the newborn in ESSC. This is the first study that provides risk estimates for CREs in neonates during ESSC. Future studies, with sufficient sample size, could consider the development of a predictive model that integrate the factors identified in this study with other candidate factors associated with SUPC. These studies could be performed on different populations, such as newborns in ESSC versus newborns in cradle and vaginal delivery with epidural analgesia versus that without epidural analgesia. The performance of the risk score should, subsequently, be implemented and evaluated to estimate its impact in perinatal care before issuing specific clinical practice recommendations.

The interpretation of the study results requires to consider some limitations, such as the difficulty in generalizing them to other populations who could benefit from ESSC. As already mentioned in this study, only HTNs with normal births and without any added risk have been included. In most centers, children born after instrumental delivery or with some type of added risk, whether maternal or child related, also undergo ESSC. This group of newborns have not been included in our study. Moreover, groups such as late preterm newborns and neonates diagnosed with intrauterine malformations that do not contraindicate ESSC have not been assessed. The identified prognostic factors should be assessed in other clinical settings, e.g., different level of hospital care. Additionally, it has not been possible to evaluate in this study some known or suspected risk factors of SUPC, such as the first breastfeeding, newborn position or ambient and axillar newborn temperature.

Among the strengths of the study, it is noteworthy to highlight that it focuses on the largest newborn population, HTNs, who receive ESSC, currently a standard practice. The multicenter, multiregional features of the study contribute to facilitate the generalizability of its findings to healthy newborns. The original design of the study, a clinical trial, and the prospective data collection increase the data quality and the confidence in its results.

## Conclusion

Cardiorespiratory events occur in two-thirds of HTNs during ESSC. Maternal age ≥35 years, gestational BMI >25 kg/m^2^, maternal primiparity, and birth during nighttime are independent predictors of CREs during ESSC. These variables are easy to evaluate and measure in standard practice. These prognostic factors can be used to identify a group of neonates at higher risk and aid healthcare professionals and parents in decision-making in the immediate postnatal period. Higher-risk HTNs might benefit from increased surveillance by parents or professionals, and from pulse oximeter monitoring during ESSC. Further studies are needed to confirm the prognostic performance of these predictors in routine clinical practice and to assess its impact in perinatal care improvement.

## Data Availability Statement

The raw data supporting the conclusion of this manuscript will be made available by the authors, without undue reservation, to any qualified researcher whose methodologically sound proposal has been approved by the Scientific Coordinator Committee for this clinical trial and with a signed data access agreement.

## Ethics Statement

The studies involving human participants were reviewed and approved by the Clinical Research Ethics Committee of all participating centers. Written informed consent to participate in this study was provided by the participants’ legal guardian/next of kin.

## Author Contributions

CP-A conceptualized and designed the study, contributed to interpreting the results, and drafted and critically reviewed the manuscript. JR-L contributed to the design of the study and acquisition of data, organized the database, coordinated and supervised data collection, contributed to the data analysis and interpreted the results, and drafted and critically reviewed the manuscript. JD performed the statistical design and analysis, contributed to the interpretation of the results, drafted and critically reviewed the manuscript, and takes primary responsibility for communication with the journal and editorial office during the submission process, throughout the peer review and during publication. NG-L contributed to the design of the trial and acquisition of data and critically reviewed the manuscript. AP, LR, FC, MV, and ÓG-A conceptualized and designed the study, contributed to the interpretation of the results, and critically reviewed the manuscript. IA, NM, AP-L, PL, AM-A, CC, EG, and IO contributed to acquisition of data and critically reviewed the manuscript. All authors approved the final manuscript as submitted and agreed to be accountable for all aspects of the work.

## Conflict of Interest

The authors declare that the research was conducted in the absence of any commercial or financial relationships that could be construed as a potential conflict of interest.

## Publisher’s Note

All claims expressed in this article are solely those of the authors and do not necessarily represent those of their affiliated organizations, or those of the publisher, the editors and the reviewers. Any product that may be evaluated in this article, or claim that may be made by its manufacturer, is not guaranteed or endorsed by the publisher.

## Footnotes

1
http://www2.redsamid.net/login.php
